# SHMT2 Overexpression Predicts Poor Prognosis in Intrahepatic Cholangiocarcinoma

**DOI:** 10.1155/2018/4369253

**Published:** 2018-08-28

**Authors:** Shanglei Ning, Siquan Ma, Abdul Qahar Saleh, Lingyu Guo, Zixiao Zhao, Yuxin Chen

**Affiliations:** ^1^Department of General Surgery, Qilu Hospital, Shandong University, Jinan, China; ^2^Department of General Surgery, Heze Second People's Hospital, Heze, China

## Abstract

**Background and Objective:**

Serine hydroxymethyltransferase 2 (SHMT2) functions as a key enzyme in serine/glycine biosynthesis and one-carbon metabolism. Recent studies have shown that SHMT2 participated in tumor growth and progression in a variety of cancer types. The objective of the present study is to explore the expression of SHMT2 and evaluate its prognostic value in patients with intrahepatic cholangiocarcinoma (iCCA).

**Patients and Methods:**

We retrospectively investigated the expression of SHMT2 in 100 primary iCCA samples through immunohistochemical (IHC) staining on a tissue array.

**Results:**

High SHMT2 expression was found in 52 of the 100 specimens. The results indicated that SHMT2 level was upregulated compared to adjacent nontumor intrahepatic bile duct tissue. Furthermore, SHMT2 level was closely associated with tumor T stage (*P* = 0.017) and tumor TNM stage (*P* = 0.041) in patients with iCCA, but not with age, gender, tumor size, tumor number, pathological grade, vascular invasion, or N stage. Moreover, Kaplan-Meier analysis suggested that patients with lower SHMT2 level have longer survival rate than those with high expression (45.8 vs 23.1%, *P* = 0.030). Additionally, the multivariate analysis model indicated SHMT2 is an independent adverse prognosticator in iCCA.

**Conclusion:**

High SHMT2 level was correlated with poorer overall survival in patients with iCCA. SHMT2 was proved to be a powerful and independent prognostic factor and a potential therapeutic target for patients with iCCA.

## 1. Introduction

Intrahepatic cholangiocarcinoma (iCCA) is the second most common primary hepatic malignancy, with an increasing incidence rate reported worldwide. Currently, surgical resection is potentially the only curative therapeutic option available. However, the majority of patients present with advanced stage disease owing to no obvious symptoms [1], resulting in a very low resection rate, and poor prognosis of patients with iCCA [2, 3]. Conventional chemotherapy and radiotherapy yield unsatisfactory results; therefore, identification of potential biomarkers for the early diagnosis of iCCA is needed urgently.

Serine hydroxymethyltransferase (SHMT) functions as a crucial enzyme in the serine/glycine synthesis pathway and one-carbon metabolism, which provides the essential precursors for protein and nucleic acid synthesis for cancer growth and metastasis [4]. Two types of SHMT genes have been discovered in the human genome, namely, SHMT1 and SHMT2. SHMT2 is located in the 12q13 chromosomal loci [5] and encoded a 55 kD protein, which existed predominantly in the mitochondrion, whereas SHMT1 encoded cytoplasmic isozymes which can be transported to the nucleus. Recent studies have shown that SHMT2 expression has increased significantly in various types of cancer and correlates with poor prognosis [6–10]. SHMT2 knockdown in hepatocellular cancer cell lines was found to reduce cell growth and tumorigenicity in vitro and in vivo [6].

Gene set enrichment analysis found that SHMT2 was significantly associated with cancer invasion and poor survival among breast cancer patients [7]. In glioma patients, SHMT2 had increased, and its expression was an independent prognostic factor [8]. However, the expression of SHMT2 in iCCA and its relationship with prognosis have not been reported. The present study was conducted to explore SHMT2 expression in iCCA and evaluate its prognostic significance.

## 2. Materials and Methods

### 2.1. Patients and Tissue Samples

We used a retrospective population-based outcome strategy to analyze specimens from 128 iCCA patients who were diagnosed in the Qilu Hospital of Shandong University, between May 2005 and May 2015. Finally, 100 patients who complied with the following criteria were included in the present study: radical tumor resection (R0), no chemotherapy or radiation prior to or after surgery, no several perioperative complications that affects survival time, and follow-up with intact information. Demographic data and neoplasm characteristics are summarized in Table 1. The last follow-up for this study was in December of 2016, and the median follow-up period of patients was 37 months (from 5 to 115 months). All the hematoxylin/eosin-stained (HE) sections were reviewed by two pathologists, according to the diagnostic criteria of the International Union Against Cancer (8th edition). The study was approved by the Ethics Committee of Qilu Hospital, Shandong University. Patients were informed, and their consent was obtained for this study.

### 2.2. Tissue Microarray Construction and Immunohistochemical Staining

Tumor specimens were obtained from the Department of Pathology, Qilu Hospital of Shandong University. HE staining was used to guide typical tumor areas from a total of 100 formalin-fixed, paraffin-embedded iCCA blocks, and 3 random representative 0.6 mm tissue cores were punched from each case and inserted into a recipient block using a tissue array (Beecher Instruments, Silver Spring, MD). The tissue array was cut 5 *μ*m thick and stained with anti-SHMT2 as the primary antibody (cat. no EPR3198, rabbit monoclonal, 1:100; Abcam, Cambridge, MA, USA). Shortly, the slides were deparaffinized and pretreated in citrate buffer (pH 6.0). Endogenous peroxidase activity was blocked with 3% hydrogen peroxide. Then, the slides were incubated with anti-SHMT2 (dilution 1 : 100) antibody working solution overnight at 4°C. After several washes, the sections were incubated with secondary antibodies for 30 min at 37°C. Finally, immunoreactivity was detected by the DAB staining kit. BSA was applied for quality control.

### 2.3. Evaluation of SHMT2 Expression

The immunoreactivity of SHMT2 in the mitochondria was scored according to the intensity and proportion of positively stained cells, as described previously. The slides were evaluated using light microscopy by two independent pathologists blinded to the clinicopathological data of patient samples. The staining intensity was scored as 0 (negative), 1 (weak), 2 (moderate), or 3 (strong). The percentage of stained cells was defined as follows: 1 (0–20% positive), 2 (21–50% positive), 3 (51–75% positive), and 4 (76–100% positive). The final IHC score (IHCS) was calculated by multiplying the intensity score by the percent score (range = 0–12). The ROC curve is used to determine 4 as the cutoff value for SHMT2 staining, and SHMT2 expression was divided into two subgroups: low and high.

### 2.4. Western Blotting

Protein extracted from 6 tumor and paired nontumor tissues was analyzed for SHMT2 protein levels by Western blotting. Immunoblotting was blocked in 5% BSA for 1 h and incubated with antibody against SHMT2 at 4°C overnight. After TBST washing, the membrane was incubated with horseradish peroxidase- (HRP-) conjugated secondary antibody for another 2 h at room temperature. Pierce's enhanced chemiluminescent (ECL) was used for detecting immunoreactivity.

### 2.5. Statistical Analysis

Statistical analysis was performed using SPSS 19.0 software (SPSS Inc., Chicago, IL, USA). Correlations between SHMT2 expression and clinicopathological parameters were determined by chi-squared and Fisher's exact tests. The survival rates were calculated by Kaplan-Meier method curves and compared using the log-rank test. The significance of prognostic factors was evaluated through a multivariate Cox proportional hazard regression. *P* value less than 0.05 was considered statistically significant.

## 3. Results

### 3.1. Patients' Characteristics and SHMT2 Expression in iCCA Tissues

**Figure 1:**
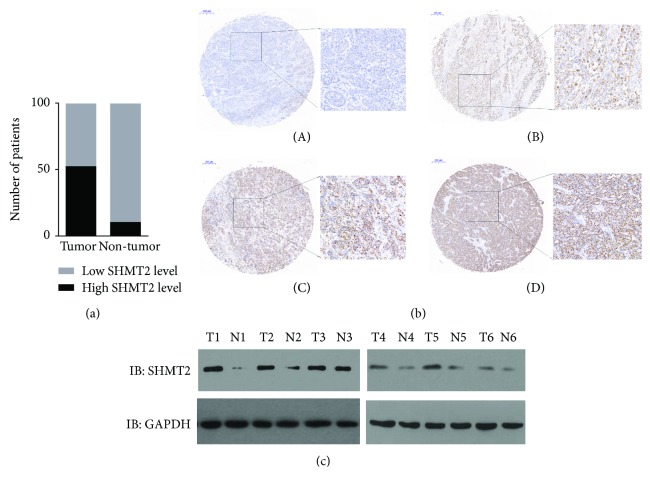
(a) SHMT2 expression is higher in tumor than in paired nontumor tissue. IHC score results demonstrated that high SHMT2 expression was observed in 52 of the 100 tumor specimens while only 15 of the paired nontumor tissue showed SHMT2 high expression. (b) SHMT2 expression patterns in intrahepatic cholangiocarcinoma. A: negative staining (−), B: weakly positive staining (+), C: positive staining (++), and D: strongly positive staining (+++). Round photographs represent TMA cores, and quadrate photographs represent specific areas from TMA cores at magnification 200x. (c) SHMT2 protein level is significantly higher in representative tumor tissue than in paired nontumor tissue. Representative Western blot images from three independent experiments are shown. T means tumor and N means paired nontumor.

We enrolled 100 iCCA patients in our study. The median patient age was 61 years (ranging between 31 and 81), 69 of which were male. The mean tumor diameter was 4.2 cm (range 2.4–10.7 cm). Overall, 89 cases were staged with T1–T2, while the other 11 cases were staged as T3–T4 at the time of iCCA tumor resection. Thirty-six cases showed vascular invasion, out of which 14 patients suffered from positive lymph node (LN) metastasis. Totally, 91 cases had solitary tumor focus; the remaining 9 patients had multiple tumor foci. As for the TNM stage, 34 patients were staged as stage I; at the time of operation, 42 patients were staged as stage II; lastly, 24 patients were staged as stage III and stage IV. The IHC staining results showed that SHMT2 was primarily expressed in the cytoplasm in iCCA. High SHMT2 expression was observed in 52 tumor specimens of the total 100 cases, according to previous criteria. Meanwhile, only 15 of the paired nontumoral tissue showed SHMT2 high expression (Figure 1(a)). Representative immunohistochemical staining for SHMT2 is shown in Figure 1(b). In addition, we detected SHMT2 expression in 6 paired tumor and nontumor tissues using Western blotting. The results showed that SHMT2 level was notably increased in tumor samples compared to paired controls (Figure 1(c)).

### 3.2. Correlation between SHMT2 Expression and Clinicopathological Features in iCCA

We then analyzed the relationship between SHMT2 expression and the clinicopathological parameters of iCCA (Table 1). The results showed that SHMT2 expression is higher in patients staged as T3–T4 (81.82%, 9/11) than those staged as T1 (38.10%, 16/42) and T2 (57.44%, 27/47) (*P* = 0.017). Similarly, we found that SHMT2 high expression was correlated with TNM stage significantly (*P* = 0.041). However, there is no significant difference in relationships between SHMT2 expression, gender, age, tumor size, tumor number, lymph node metastasis, vascular invasion, or pathological grade.

### 3.3. SHMT2 Is an Independent Prognostic Biomarker in iCCA

**Figure 2:**
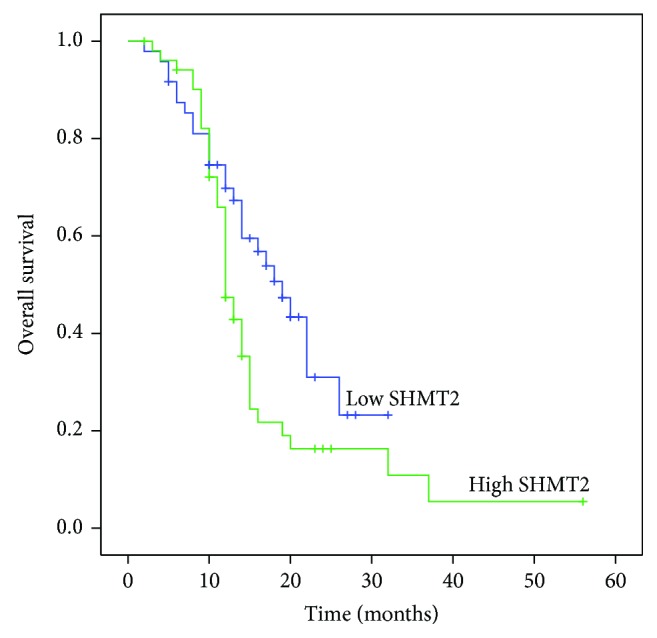
Overall survival curves of patients for intrahepatic cholangiocarcinoma with different SHMT2 expression levels. Patients with low expression of SHMT2 have a significantly better survival rate than those with high SHMT2 expression (*P* = 0.030).

A Kaplan-Meier analysis showed that patients with higher SHMT2 expression had lower survival rates than those with lower SHMT2 expression (23.1 vs 45.8%, *P* = 0.030; Figure 2). Besides, univariate analysis demonstrated that tumor number (35.2 vs 22.2%, *P* = 0.012) and T stage (40.5 vs 34.0 vs 9.0%, *P* < 0.001) were closely associated with the overall survival of patients with iCCA (Table 2). Then, multivariate analysis was performed using the Cox proportional hazard model for all of the significant variables, which were examined in the univariate survival analysis (Table 3). In addition to T stage, we found that SHMT2 is an independent prognostic factor for iCCA (*P* = 0.021, CI = 1.168–6.453).

## 4. Discussion

ICCA is a rare but highly fatal gastrointestinal neoplasma, which demonstrated distinct clinical and biological features from hilar and distal cholangiocarcinoma [11]. It accounts for about 10% of all cholangiocarcinoma and 10–20% of liver cancer [12, 13]. The incidence and mortality of iCCA have been increasing worldwide [14, 15]. Surgical resection is the only option that offers long-term survival possibility. Although adjuvant chemotherapy, radiotherapy, and even target drugs have been applied in clinical course, unfortunately, its prognosis was still very poor, owing to nonspecific symptom and delayed clinical diagnosis at an advanced stage [3, 4].

SHTM2 is a key metabolic enzyme which is involved in the conversion from serine to glycine and folate cycle. Its gene maps to 12q13 [5] and encodes a protein that localized to the mitochondria predominately [16], while its isoform SHMT1 is mainly expressed in the cytoplasm. Long considered as a housekeeping biological procedure, serine and one-carbon metabolism provides raw material for the synthesis of nucleotides and amino acids; moreover, it serves as a source of methyl groups in molecular methylation [17, 18]. Although SHMT2 inhibitors have not been applied in cancer therapy, some chemotherapy drugs targeting SHMT2 downstream enzymes, in one-carbon metabolism such as 5-FU and gemcitabine, have significantly shown a dramatic response in subsets of cancer patients [19]. Recently, Ducker et al. revealed that a small molecule SHMT inhibitor could block the growth of many types of human cancer cells through decreasing glycine import, especially in B cell lymphoma [20]. Highly qualified oncogenomics have identified SHMT2 as a potential cancer driver gene [9]. Moreover, recent studies have shown that SHMT2 expression level is generally higher than normal tissues in a variety of cancer and it plays crucial roles in cancerous cell growth and aggressiveness [6–10]. Clinical data demonstrates that elevated expression of SHMT2 was shown to be independently associated with PFS and RFS [21, 22]. However, the expression of SHMT2 in iCCA and its correlation with clinicopathological features have not been elucidated.

The inclusion criteria eliminated the bias of survival analysis due to palliative therapy, R1 resection, or severe complication after surgery. The data of the current study showed that SHMT2 level was increased compared to nontumoral bile duct tissue. Furthermore, SHMT2 expression is significantly related with tumor T stage (*P* = 0.017) and tumor TNM stage (*P* = 0.041) in patients with iCCA, disregarding age, gender, tumor size, tumor number, pathological grade, vascular invasion, or lymph node metastasis. With the increase in TNM stage, the expression of SHMT2 increased. The results demonstrated that serine and glycine metabolism increased in more advanced intrahepatic cholangiocarcinoma cells.

A Kaplan-Meier analysis suggested that patients with lower SHMT2 level have a better overall survival rate. In addition to the tumor number and T stage, multivariate analysis and the Cox proportional hazard regression model revealed that SHMT2 expression is an independent prognostic factor for patients with iCCA, consistent with previous SHMT2 clinical reports in other solid cancers [6–8, 23]. Consistent with the results, Lee et al. took advantage of RNAi loss of function screen targeting 620 candidate genes across 32 cell lines and identified SHMT2 necessary for tumor survival [9]. However, the molecular mechanism, through which SHMT2 affects tumor proliferation and prognosis, remains unclear. Interestingly, Zhang et al. found that glycine decarboxylase (GLDC) was upregulated in SHMT2 overexpressed 3T3 cells and whether or not GLDC is responsible for the cell proliferation induced by SHMT2 remains to be explored [24]. Besides, Kim et al. found that SHMT2 drives glioma cell survival under a hypoxic environment through regulating the activity of pyruvate kinase negatively and inhibiting carbon flux into the TCA cycle [25]. To our knowledge, this is the first report to indicate the oncogenic role of SHMT2 in iCCA. Although this study enrolled a limited number of patient samples, these data may help elucidate the biological functions of SHMT2 and its molecular mechanisms in the disease.

In conclusion, this study describes higher SHMT2 expression and its independent prognostic value in patients with iCCA. Furthermore, the data demonstrated a correlation between SHMT2 expression and tumor aggressiveness (TNM stage) in iCCA. SHMT2 may become a potential prognostic biomarker and molecular therapy target for the treatment of patients with iCCA. We intend to investigate the exact mechanism of SHMT2 on prognosis in future studies.

## Figures and Tables

**Table 1:** Relationship between SHMT2 expression and conventional clinicopathological parameters in human intrahepatic cholangiocarcinoma.

Clinicopathological parameters	*n*	SHMT2 expression		*P* ^∗^
		Low	High	
Gender				0.959
Female	31	15	16	
Male	69	33	36	
Age (years)				0.987
<60	52	25	27	
≥60	48	23	25	
Tumor size (cm)				0.935
≤5	40	19	21	
>5	60	29	31	
Tumor number				0.351
Solitary	91	45	46	
Multiple	9	3	6	
Vascular invasion				0.120
No	64	27	37	
Yes	36	21	15	
Pathological grade				0.361
I	4	3	1	
II	62	27	35	
III	34	18	16	
T stage				0.017^∗^
T1	42	26	16	
T2	47	20	27	
T3–T4	11	2	9	
N stage				0.318
N0	86	43	43	
N1	14	5	9	
Metastasis				0.261
No	96	45	51	
Yes	4	3	1	
TNM stage				0.041^∗^
I	34	22	12	
II	42	18	24	
III–IV	24	8	16	

**Table 2:** Univariate analysis of clinicopathological features for overall survival of patients with intrahepatic cholangiocarcinoma.

Characteristics (*n* = 100)	Number	Survival rate (%)	*P* ^∗^
Gender			0.064
Female	31	32.3	
Male	69	34.8	
Age (years)			0.325
<60	52	38.5	
≥60	48	29.2	
SHMT2 expression			0.030^∗^
Low	48	45.8	
High	52	23.1	
Vascular invasion			0.470
No	64	31.3	
Yes	36	38.9	
Tumor number			0.012^∗^
Solitary	91	35.2	
Multiple	9	22.2	
Tumor size (cm)			0.484
≤5	40	32.5	
>5	60	35.0	
Pathological grade			0.800
I	4	50	
II	62	33.9	
III	34	32.4	
T stage			<0.001^∗^
T1	42	40.5	
T2	47	34.0	
T3–T4	11	9.0	
N stage			0.888
N0	86	33.7	
N1	14	35.7	
Metastasis			0.073
No	96	35.4	
Yes	4	0	
TNM stage			0.058
I	34	38.2	
II	42	35.7	
III–IV	24	25.0	

**Table 3:** Multivariate analysis (Cox regression) of clinicopathological characteristics of 100 patients with intrahepatic cholangiocarcinoma.

Variable	Category	*P*	HR	95% CI
SHMT2 expression	Low			
	High	0.021	2.746	1.168–6.453
Tumor number	Solitary			
	Multiple	0.114	1.937	0.854–4.394
T stage	T1	0.001	0.258	0.115–0.579
	T2			
	T3 T4			

Abbreviations: HR = hazard ratio; CI = confidence interval.

## Data Availability

The data used to support the findings of this study are available from the corresponding author upon request.
